# OverlappING and DivergING roles of ING4 and ING5 in cancer, differentiation and development

**DOI:** 10.3389/fcell.2026.1850852

**Published:** 2026-07-27

**Authors:** Sergey Dadoyan, Tom J. Bartizal, Sakunika Amarsingha, Karl Riabowol

**Affiliations:** 1 Robson DNA Sciences Centre, Arnie Charbonneau Cancer Institute, University of Calgary, Calgary, AB, Canada; 2 Department of Biochemistry and Molecular Biology, University of Calgary, Calgary, AB, Canada; 3 Cumming School of Medicine, University of Calgary, Calgary, AB, Canada; 4 The Calvin, Phoebe and Joan Snyder Institute for Chronic Diseases, University of Calgary, Calgary, AB, Canada; 5 Faculty of Veterinary Medicine, University of Calgary, Calgary, AB, Canada; 6 Alberta Children’s Hospital Research Institute, University of Calgary, Calgary, AB, Canada; 7 Arthur JE Childs Comprehensive Cancer Institute, University of Calgary, Calgary, AB, Canada; 8 Department of Oncology, University of Calgary, Calgary, AB, Canada

**Keywords:** acetylation, chromatin, epigenetic, histone, ING proteins, paralog, stem cells

## Abstract

The INhibitor of Growth (ING) proteins are plant homeodomain (PHD) zinc-finger epigenetic readers that recognize trimethylated lysine 4 of histone H3 (H3K4me3) and couple it to lysine-acetyltransferase (KAT) or -deacetylase (KDAC) activity on chromatin. ING4 and ING5 are among the family’s closest paralogs, which share a four-domain architecture and high sequence identity, with divergence concentrated outside the PHD finger. Earlier reviews focused either on the family as a whole or on the individual INGs. This paper is the first to directly compare ING4 and ING5, including their structures, functions within KAT complexes, and roles in cancer and development, noting where they overlap and diverge. Either paralog can occupy the ING position in HBO1 (KAT7) complexes, whereas only ING5 is found in MOZ and MORF (KAT6A/B) complexes. In cancers, ING4 and ING5 generally act as tumor suppressors but can also promote malignancy and stemness. The bulk of evidence for mechanistically explaining the effects of ING4/5 in cancers comes from ectopic overexpression studies. In differentiation, ING5 has been investigated far more than ING4, and the roles of the two mostly diverge: ING5 participates in maintaining stemness in neural, epidermal, and brain tumor initiating cell lines, as well as proliferation of mesenchymal stem cells, whereas ING4 was found to restrain hematopoietic stem cell self-renewal, promote prostate epithelial differentiation, but promote stemness of renal cell carcinoma cells. They nonetheless compensate for one another in embryonic survival, since either paralog alone prevents the arrest seen in double knockouts. The key remaining questions include what determines the structural basis of ING5’s MOZ/MORF selectivity, what the precise conditions are under which they substitute for one another, how exactly ING4 and ING5 can be targeted in cancer therapy, and whether the roles of the two paralogs are conserved across species.

## 1 Introduction

**FIGURE 1:**
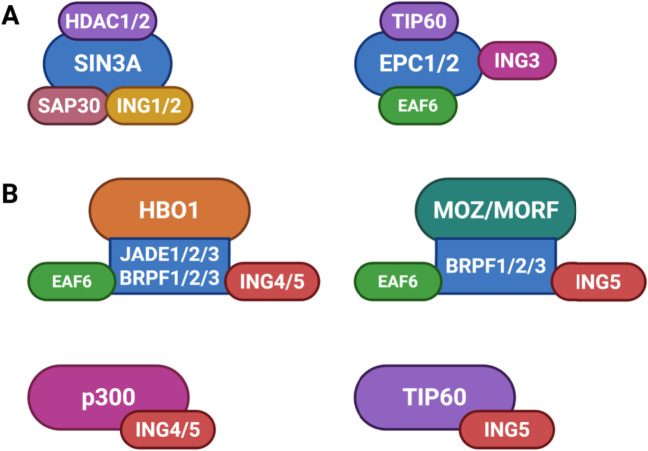
ING family proteins are core components of KAT and KDAC complexes. **(A)** ING1-3 major complexes. ING1 and ING2 are subunits of HDAC1/2 complexes while ING3 is a subunit of the TIP60 KAT complex. **(B)** ING4-5 complex memberships and known physical interactions with KATs. Top row: ING4 and ING5 have both been shown to associate with the HBO1 complex independently. JADE and BRPF act as scaffolding subunits, linking EAF6 and either ING4 or ING5 to the relevant KAT protein. Scaffold association has been shown to alter KAT histone preference: BRPF drives H3 acetylation, while JADE directs complex activity towards H4. ING5 also associates with the MOZ/MORF (KAT6A/B) complex and is directed towards H3 acetylation through BRPF that acts as a core member of these complexes. Bottom row: ING4 and ING5 were reported to physically interact with p300 and ING5 was reported to interact with TIP60.

The INhibitor of Growth (ING) family of proteins was first identified in 1996 with the discovery of ING1 (Garkavtsev et al., 1996). INGs are Plant Homeodomain (PHD) finger epigenetic readers that bind the H3K4me3 mark of active transcription. They act as subunits in the KAT or KDAC complexes that bridge H3K4me3 recognition with the local acetylation or deacetylation of chromatin (Loewith et al., 2000; Doyon et al., 2006; Peña et al., 2006; Shi et al., 2006; Champagne et al., 2008; Palacios et al., 2008; Gatchalian et al., 2016; Kim et al., 2016). Each ING was found to form distinct chromatin-modifying complexes: ING1 and ING2 with the histone deacetylases 1/2 (HDAC1/2) KDACs (Skowyra et al., 2001; Kuzmichev et al., 2002; Doyon et al., 2006), and ING3-ING5 with the MOZ, YBF2/SAS3, SAS2, TIP60 (MYST)-family KATs: TIP60 (with ING3), HBO1 (with ING4 and ING5), and MOZ/MORF (with ING5) (Doyon et al., 2004; Doyon et al., 2006). In addition, ING4 and ING5 were reported to physically interact with p300 (EP300) (Shiseki et al., 2003), and ING5 was demonstrated to function as a TIP60 (KAT5) cofactor in the context of promoting p53 (TP53) acetylation (Liu et al., 2013) (Figure 1).

**FIGURE 2:**
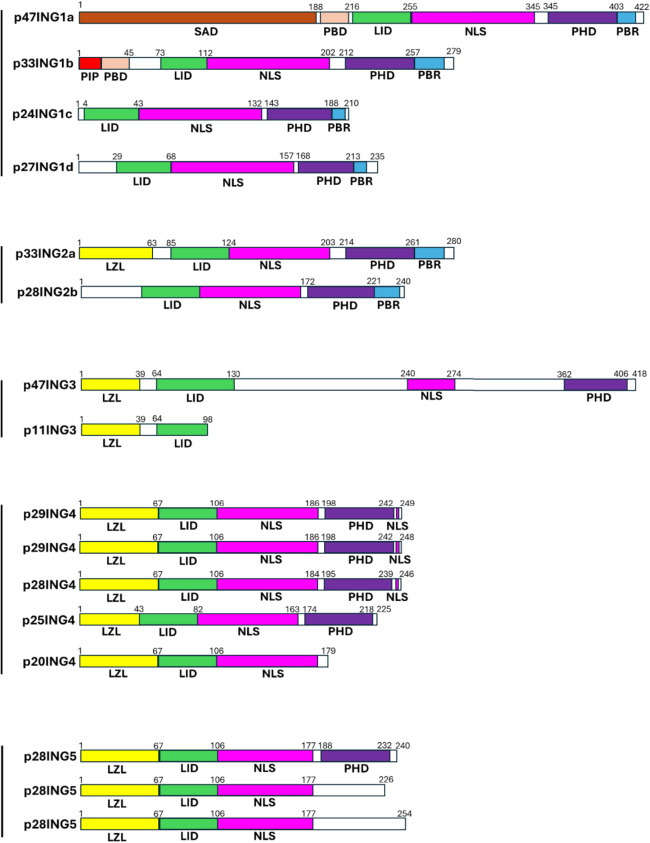
Domains of human ING1-5 family isoforms. Each ING protein family member has several isoforms produced via alternative splicing or alternative promoter usage. Each contains conserved functional domains critical for the functions of the INGs. The PHD in all INGs facilitates binding to the H3K4me3 histone mark (Champagne and Kutateladze, 2009). The LID promotes lamin-A interactions with ING1 to maintain nuclear morphology (Han et al., 2008). The NLS contains basic Nucleolar Targeting Sequences (NTSs), promoting ING translocation to the nucleus and nucleolus (Scott et al., 2001; Unoki et al., 2006). The LZL region facilitates ING2-dependent muscle cell differentiation of C2C12 myoblasts (Eapen et al., 2012). ING1 and ING2 have polybasic region (PBR) motifs, aiding in phosphoinositide (Gozani et al., 2003) and ubiquitin (Thalappilly et al., 2011) binding. Only p33ING1b and p47ING1a contain a partial bromodomain (PBD), which interacts with the 30 kDa Sin3A-associated protein (SAP30) (Kuzmichev et al., 2002). P33ING1 additionally has a PCNA-interacting Protein Motif (PIP) which participates in the UV-induced DNA damage response (Scott et al., 2001). The p47INGa protein contains a senescence-associated domain (SAD) known to target mitochondria and induce senescence (Bose et al., 2013; Bertschmann et al., 2026).

While all INGs share many structural similarities (Figure 2), ING4 and ING5 are the closest paralogs and exhibit >67% overall amino-acid sequence identity (He et al., 2005; Bertschmann et al., 2026). Both proteins associate with HBO1 (KAT7) and can homo- and heterodimerize via N-terminal coiled-coil contacts (Culurgioni et al., 2012; Ormaza et al., 2019). At the functional level, they are mainly associated with tumor suppressor roles (Tallen and Riabowol, 2014; Dantas et al., 2019; Du et al., 2019; Zheng et al., 2022a; Liao et al., 2025). However, potential pro-malignant properties such as the ability to maintain stemness in cancer cells (Wang et al., 2018; Tang et al., 2022) and regulation of stemness in somatic cells and in embryonic development (Mulder et al., 2012; Berger et al., 2014; Wang et al., 2018; Cenik et al., 2022; Kim et al., 2023; Mah et al., 2023; Mah et al., 2024; Al-Shueili et al., 2025) have been described.

Prior reviews of the ING family fall into three categories. The most extensive survey all five paralogs (Feng et al., 2002; Coles and Jones, 2009; Archambeau et al., 2019) while single-paralog reviews for every ING cover their roles in various contexts and one review directly describes and compares ING1 and ING2 (Guérillon et al., 2013), another evolutionarily and functionally closely related pair in the ING family (He et al., 2005; Bertschmann et al., 2026). In this review we ask where ING4 and ING5 can functionally substitute for one another, and how their ∼33% amino acid sequence divergence translates into paralog-specific biology. We focus on domain architecture, KAT complex components, mechanisms in cancer, differentiation and development, with an emphasis on compensation or divergence between these very similar paralogs.

## 2 Sequence and structure of ING4 and ING5

**FIGURE 3:**
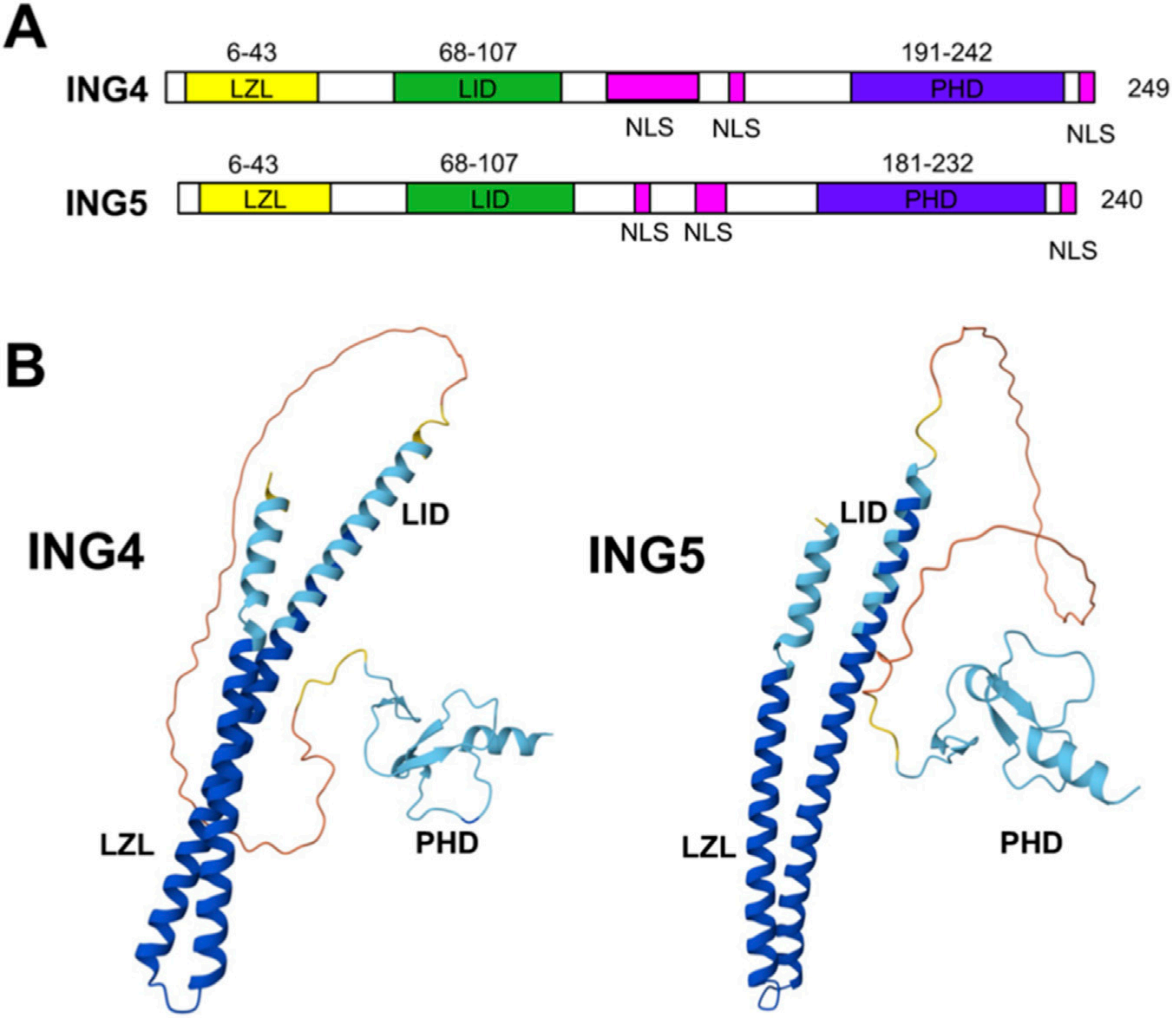
Domains and predicted structure of ING4 and ING5. **(A)** Domains of the major 249 aa ING4 and 240 aa ING5 isoforms. **(B)** The structures of ING4 (AF-Q9UNL4-4-F1-v6) and ING5 (AF-Q8WYH8-F1-v6) predicted by AlphaFold three indicate that the LZL and LID form helices, that the PHD regions are highly structured and that these structured regions are separated by the highly unstructured central regions of the proteins indicated by the red lines that contain NLS regions. AlphaFold pLDDT scores for ING4 and -5 predictions are listed as 77.5% and 78.62%, respectively. pLDDT >70, but <90, are described as indicating a well modelled protein backbone, but caution should be exercised when applying this to processes that require high degrees of accuracy such as binding site predictions.

ING4 and ING5 share a four-element modular architecture (Figure 3) that includes an N-terminal leucine zipper-like (LZL) domain, a lamin-interacting domain (LID, previously called a novel conserved region or NCR), a central basic region containing a nuclear localization sequence (NLS) that is largely disordered, and a well conserved C-terminal PHD finger. (He et al., 2005; Soliman and Riabowol, 2007; Han et al., 2008; Ormaza et al., 2017; Ormaza et al., 2019). A second NLS domain was identified in ING4 and predicted in ING5 (Unoki et al., 2006; Soliman and Riabowol, 2007).

### 2.1 The LID domain

The LID is unique to the ING family within the human proteome. In ING1, the LID mediates direct binding to lamin A, mapped to the lamin A rod domain (residues 1–406); disruption of this interaction in fibroblasts induced nuclear morphological defects reminiscent of laminopathies such as Hutchinson-Gilford progeria syndrome, and reduced ING1-induced apoptosis. The LID sequence is conserved across ING1-5, and in lamin A co-immunoprecipitations from HEK293 cells expressing each paralog, ING1-4 were recovered to varying degrees with ING4 showing the strongest band after ING1 but ING5 was not detected (Han et al., 2008). Whether ING4-lamin A association produces functional consequences analogous to those seen for ING1 has not been tested.

### 2.2 The NLS domain

The NLS promotes nuclear import via the importin α/β pathway (Chook and Blobel, 2001; Scott et al., 2001). ING4 contains a bipartite NLS with two basic clusters (KK and RARSK); the RARSK cluster is required for nuclear localization and for direct p53 binding *in vitro* and in MCF-7 cells (Zhang et al., 2005). A second predicted basic motif (NLS2) in ING4 is dispensable for nuclear import, since its deletion does not alter ING4 subcellular distribution (Unoki et al., 2006). The central NLS-containing region is disordered in both ING4 and ING5, tethers the PHD finger to the N-terminal coiled-coil with high conformational flexibility, and binds dsDNA with low micromolar affinity, which has been proposed to support recruitment of ING4/5-associated KATs to chromatin (Ormaza et al., 2017; Ormaza et al., 2019).

### 2.3 The PHD domain

The highly conserved PHD consists of a zinc finger with a Cys_4_-His-Cys_3_ coordination, which allows for binding two molecules of Zn^2+^ (Champagne and Kutateladze, 2009). The PHD fingers from ING4 and ING5 display the typical ING PHD fold and interact with H3K4me3 via a cage composed of two aromatic residues (Champagne et al., 2008; Palacios et al., 2008; Champagne and Kutateladze, 2009). The ING PHD fingers exhibit selectivity for H3K4me3 over less methylated H3 marks, with binding constants in the low micromolar range (He et al., 2005; Li et al., 2006; Peña et al., 2006; Kim et al., 2016). The binding of the PHD domains of ING4 and ING5 to H3K4me3 increases KAT-acetylation-mediated active transcription (Doyon et al., 2006). Binding between the ING4 PHD and the H3K4me3 target site includes fewer hydrogen bonds than those observed in other ING complexes, including ING5, which may be a factor in a slightly weaker binding affinity of ING4’s PHD (Champagne and Kutateladze, 2009).

### 2.4 The LZL domain

The N-terminal region of ING4 associated with the LZL domain folds into an antiparallel left-handed coiled-coil that assembles ING4 into a homodimer; monomeric ING4 mutants still bind HBO1 but no longer induce apoptosis in response to DNA damage and show reduced suppression of anchorage-independent cell growth, indicating that dimerization itself is required for tumor-suppressive activity independent of HBO1 association (Culurgioni et al., 2012). The N-terminal domain of ING5 adopts a structurally analogous coiled-coil and also homodimerizes; ING4 and ING5 share 75% sequence identity across this domain and co-immunoprecipitate when co-expressed in HEK293T cells, indicating that they can form heterodimers. On the other hand, purified N-terminal domains did not yield stable heterodimers *in vitro* (Ormaza et al., 2019). A scaffolding role for the LZL has been documented in ING2, where the region is required for assembly with HDAC1 and SIN3A and for ING2-dependent muscle cell differentiation in C2C12 myoblasts (Eapen et al., 2012). Whether the ING4 or ING5 LZL participates in scaffolding interactions beyond homo- and heterodimerization has not been examined.

## 3 Complex composition and paralog-specific assembly

ING4 and ING5 interact with several MYST-family lysine acetyltransferase (KAT) complexes. While the HBO1 complex was demonstrated to have either ING4 or ING5 as one of its subunits, the MOZ/MORF (KAT6A/B) complexes were shown to interact with ING5 but not ING4 (Doyon et al., 2006). Several reports also indicated physical interaction between ING4 and ING5 and p300 (Shiseki et al., 2003; Zhang et al., 2017), and one study demonstrated that ING5 co-immunoprecipitates with TIP60 in U2-OS cells (Liu et al., 2013). This section describes complex architecture, accessory subunit composition, and substrate specificity for each assembly. The functional consequences of these interactions are discussed in Sections 3, 4.

### 3.1 HBO1 complex

**FIGURE 4:**
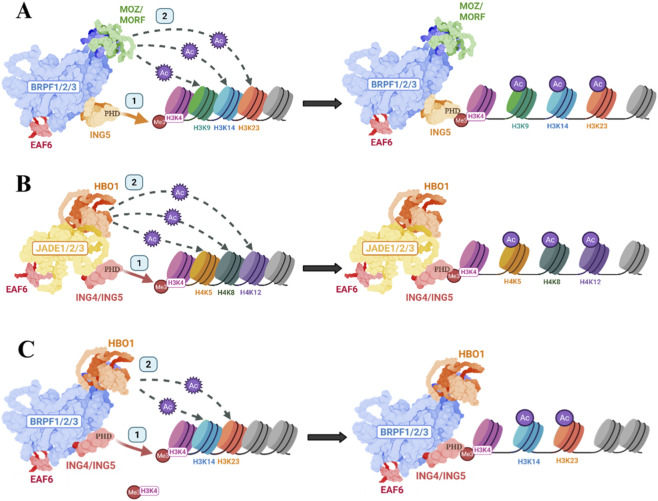
Major KAT complexes that associate with ING4/5 and acetylate sites on core histones H3 and H4 to relax chromatin structure. **(A)** ING5 binds H3K4me3 via its PHD domain [**1**], recruiting MOZ/MORF or HBO1 KAT to H3 leading to acetylation [**2**] and opening of chromatin. ING5/MOZ/MORF/BRPF1/2/3 acetylates H3K9, H3K14, and H3K23. **(B)** ING4/5/HBO1/JADE1/2/3 acetylates H4K5, H4K8, and H4K12. **(C)** ING4/5/HBO1/BRPF1/2/3 acetylates H3K14, and H3K23.

The HBO1 acetyltransferase complex contains HBO1, Esa1-associated factor 6 (EAF6/MEAF6), an ING subunit (ING4 or ING5), and a scaffold drawn from either the JADE (JADE1/2/3) or the BRPF families (BRPF1/2/3). Scaffold choice determines histone tail specificity on chromatin: HBO1-JADE acetylates histone H4 at K5/K8/K12, whereas HBO1-BRPF acetylates histone H3 at K14/K23, with HBO1 also capable of acetylating both H3 and H4 on free histones (Doyon et al., 2006; Lalonde et al., 2013) (Figures 4B,C). HBO1-JADE-ING complexes account for the majority of histone H4 acetylation in multiple human cell lines *in vivo*. HBO1/ING5, but not ING4, also co-purifies with components of the MCM2-7 helicase. ING5 depletion produced a more severe S-phase block than HBO1 depletion, with cells entering S phase but failing to incorporate BrdU or progress to G2/M, consistent with ING5 contributing to replication through both HBO1 and, potentially, MOZ/MORF complexes (Doyon et al., 2006). HBO1’s role in DNA replication was further established by direct interaction with the origin recognition complex and a requirement for MCM loading during pre-replicative complex assembly (Iizuka and Stillman, 1999; Iizuka et al., 2005; Miotto and Struhl, 2008). Another study demonstrated that HBO1-BRPF2-ING4/5 complexes interact with MIS18BP1 and contribute to centromeric CENP-A deposition (Ohzeki et al., 2016), further highlighting the importance of the complex for cell cycle progression.

### 3.2 MOZ/MORF complexes

MOZ and MORF acetyltransferase complexes contain the catalytic subunit, EAF6, ING5, and a BRPF1/2/3 scaffold. ING4 has not been identified in any MOZ-tagged or MORF-tagged purification (Doyon et al., 2006; Ullah et al., 2008). The ING5 PHD finger selectively binds H3K4me3, directing the MOZ/MORF complexes to acetylate targeted residues on surrounding chromatin (Champagne et al., 2008; Ullah et al., 2008). However, target specificity has varied across experiments. *In vitro* studies using purification of the complex from HeLa cells indicated that the primary substrate was H3K14, with no H3K9 acetylation detected and a strong preference for H3 over H4 (Doyon et al., 2006). By contrast, *in vivo* analysis identified H3K9 acetylation as regulated by MOZ, with functional consequences for body segment identity driven by changes in *Hox* gene expression. ING5 recruitment to *Hox* loci was also dependent on MOZ (Voss et al., 2009) (Figure 4A). In leukemia samples with MOZ-CBP and MORF-CBP translocations, global H4K16 acetylation was reduced. MORF-CBP cells showed particularly low levels of H4K16ac, implicating MOZ/MORF among the H4K16-specific MYST acetyltransferases alongside MOF and TIP60 (Fraga et al., 2005). More recent work on the native full-length MORF complex in K562 cells identified H3K23 as a primary substrate. Acetylation of H3K23 by MORF was coupled to recognition of acylated H3K14 by the MORF double PHD finger (DPF) reader domain (Klein et al., 2019). The H3K23 specificity was also observed for the enok (MOZ/MORF ortholog) complex in *Drosophila* tissues (Kim et al., 2023). Together, these observations suggest that MOZ/MORF-ING5 substrate specificity depends on complex composition, substrate state (free histones versus nucleosomes versus chromatin *in vivo*), and biological context (cell line, tissue, developmental stage and malignancy). Functional consequences of MOZ/MORF-ING5 chromatin recruitment are detailed in later sections.

### 3.3 p300 and TIP60

ING4 and ING5 have also been reported to interact with p300 (both ING4 and ING5) and TIP60 (ING5 only). The binding of ING4 and ING5 to p300 enhanced p300-mediated acetylation of p53 (TP53) at K382 in RKO cells, leading to apoptosis. The interaction of ING4 with p300 resulted in stronger acetylation of p53K382 in comparison to ING5-p300 interaction (Shiseki et al., 2003). Overexpression of ING5 in a lung carcinoma cell line enhanced p300 autoacetylation at the K1555, K1558, K1560, K1647 and K1794 sites. This modification resulted in the acetylation of the p300 targets p53 at K382 and H3 at K18, leading to the upregulation of p53 target genes *P21* (*CDKN1A*) and pro-apoptotic *BAX* (Zhang et al., 2017). In U2OS cells, ING5 was found to bind TIP60 in response to DNA damage, correlating with TIP60-mediated acetylation of p53 at K120. The ING5-TIP60-p53 complex was recruited to the pro-apoptotic gene *BAX* and growth arrest-associated gene *GADD45* promoters (Liu et al., 2013). Interestingly, TIP60 primarily forms a complex with ING3, another ING family member. It guides TIP60 to H3K4me3 sites, promoting acetylation of H2A and H4 histones (Doyon et al., 2004; Doyon et al., 2006; Peña et al., 2006; Kim et al., 2016).

## 4 ING4/5 in cancer models

ING4 and ING5 have been mainly described as tumor suppressors. Loss of their tumor-suppressive functions in cancers is generally caused by reduced expression or abnormal subcellular localization, without detectable mutations (Zheng et al., 2011; Tallen and Riabowol, 2014; Guérillon et al., 2014; Du et al., 2019; Shatnawi et al., 2021a; Zheng et al., 2022a). However, ING5 also shows context-dependent activities that can even sometimes be pro-malignant, including its promotion of stemness in several cancer models (Wang et al., 2018; Tang et al., 2022). This section reviews the major mechanistic axes by pathway, with attention to convergence, divergence, and context-dependence between paralogs. Their roles and the evidence supporting them are summarized in Table 1.

**TABLE 1:** Effects of ING4 and ING5 on cancer-related mechanisms.

Axis	Paralog	Cancer/Model	Key finding (direction)	Evidence type	References
p53	ING4 and ING5	RKO (colon)	Bind p53 and p300; ING4 increased p53-K382 acetylation, ING5 stronger p21 activation (tumor-suppressive, paralog-distinct)	Overexpression + co-IP (cell line)	Shiseki et al. (2003)
p53	ING5	U2OS (bone)	shRNA knockdown reduced p53-K120 acetylation, BAX/GADD45 induction, and doxorubicin-induced apoptosis (TIP60 axis, tumor-suppressive)	Knockdown/endogenous (cell line)	Liu et al. (2013)
p53	ING4 and ING5	A549 (lung)	Ectopic expression supports the p300-p53 axis (tumor-suppressive)	Overexpression (cell line)	Zhang et al. (2017)
p53	ING5	LNCaP and PC3 (prostate) + tissue	Downregulated in tumor; overexpression raised p53/acetyl-p53 (LNCaP) and suppressed proliferation, migration, invasion in p53-null PC3 (tumor-suppressive, p53-independent)	Overexpression (cell line) + clinical	Barlak et al. (2019)
p53	ING4	LNCaP (prostate)	Knockdown altered cancer-related gene expression (RNA-seq); functional outcome undetermined	Knockdown/RNA-seq (cell line)	Shatnawi et al. (2020)
p53	ING4 and ING5	Saos-2 (bone), p53^−/−^ Mdm2^−/−^ MEFs, EBV-LCLs	EBV EBNA3C binds ING4 (66–184) and ING5 (120–240), displaces p53, and represses ING-enhanced p53/p21 transactivation and apoptosis (indirect support)	Binding/overexpression (cell line) + endogenous co-IP (LCLs)	Saha et al. (2011)
NF-κb	ING4	U87MG (glioblastoma)	Represses NF-κB; loss increased NF-κB DNA binding and targets (IL-6, IL-8, COX-2, CSF3); overexpression reduced tumor growth (tumor-suppressive)	Overexpression + antisense (cell line and *in vivo* tumors)	Garkavtsev et al. (2004)
NF-κb	ING4	U251-MG, U118-MG (glioma)	Binds promoter-bound p65, dephosphorylates Ser536, displaces p300, recruits HDAC-1, and lowers H3/H4 acetylation and H3K4me3 (tumor-suppressive mechanism)	Knockdown/null + ChIP (cell line)	Nozell et al. (2008)
NF-κb	ING4	*In vitro* (recombinant)	PHD finger acts as an E3 ligase with UbcH3, K48-polyubiquitinating p65-K62 for degradation; requires C239, NLS-independent	*In vitro* reconstitution	Hou et al. (2014)
NF-κb	ING5	ESCC (esophageal)	Overexpression reduced p65, p-AKT, and MMP-9 and reduced progression (tumor-suppressive; p-AKT decreased)	Overexpression (cell line)	Zhang et al. (2018)
NF-κb	ING5	SGC-7901 and GT-3TKB (gastric)	Overexpression increased NF-κB activity, cytokines, and chemoresistance yet suppressed proliferation/invasion (context-dependent/dual; p-AKT increased); knockdown done for non-NF-κb phenotypes only	Overexpression (NF-κB) + knockdown (other phenotypes) (cell line)	Gou et al. (2015)
NF-κb	ING4	MKN-45, MNNG-transformed gastric epithelium	Suppressed NF-κB (paralog divergence from ING5 in gastric)	Cell-line studies	Li et al. (2013), Chen et al. (2018b)
Wnt/β-catenin	ING5	A549, H1299 (lung), HCT116 (colorectal)	Promotes phospho-dependent β-catenin degradation; knockdown raised and overexpression lowered β-catenin (Ser33/37), c-MYC fell, XAV939 rescue (tumor-suppressive)	Knockdown + overexpression (cell line)	Liu et al. (2019)
Wnt/β-catenin	ING4	A549 (lung)	Overexpression lowered Wnt-1, cyclin D1, and slightly β-catenin; reduced colony formation and xenografts (unquantified blots)	Overexpression (cell line) + xenograft (*in vivo*)	Li et al. (2008a)
Wnt/β-catenin	ING4	A549 (lung)	miR-650 represses endogenous ING4, which inversely tracks Wnt-1/β-catenin; re-expression partially reverses (tumor-suppressive)	Endogenous (miR-650 modulation) (cell line)	Tang et al. (2019)
Autophagy	ING4	U2OS (bone) HCT116 (colorectal)	Endogenous ING4 represses the BECN1/PIK3C3 autophagy complex; depletion raised flux; overexpression no effect (tumor-suppressive, best-controlled)	Knockdown/endogenous (cell line)	Sica et al. (2017)
Autophagy	ING4	U251MG, LN229 (glioma)	Adenoviral overexpression triggered autophagic and apoptotic cell death (rescued by 3-MA or BECN1/ATG7 knockdown); cytotoxic feature, not pro-autophagic regulation	Overexpression (cell line)	Gong et al. (2013)
Autophagy	ING5	SGC-7901 (gastric)	Overexpression reportedly raised autophagy and BECN1 but lowered ATG7/ATG14 and raised PI3K/p-AKT; reduced apoptosis, chemoresistance; internally inconsistent, no flux assay (unresolved)	Overexpression (cell line)	Gou et al. (2015)
Cell motility (liprin α1)	ING4	RKO (colon); HEK-293 (kidney), U87MG (glioma)	Binds liprin α1 (PPFIA1) in the cytoplasm (absent for ING5); overexpression suppressed and knockdown increased spreading/migration, lost on liprin α1 knockdown (tumor-suppressive, motility)	Overexpression + knockdown + co-IP (cell line)	Shen et al. (2007)
c-MYC translation (AUF1)	ING4	K562 (leukemia), HeLa (cervix)	Binds AUF1 and represses c-MYC translation (overexpression lowers, knockdown raises c-MYC protein; mRNA and turnover unchanged) (tumor-suppressive)	Overexpression + knockdown (cell line)	Lu et al. (2013a)
HIF-1α/angiogenesis	ING4	Multiple myeloma lines	Associates with PHD2/EGLN1; knockdown raised HIF-1α DNA binding, NIP-3, and IL-8/osteopontin angiogenesis; overexpression alone insufficient (tumor-suppressive)	Knockdown (cell line)	Colla et al. (2007)
Angiogenesis (JWA/ILK)	ING4	Melanoma + patient TMA	Activates JWA, lowering ILK and ILK/IL-6/VEGF angiogenesis; low JWA and high ILK predict poorer survival (tumor-suppressive)	Mechanistic (cell line) + clinical (TMA)	Lu et al. (2013b)
Stemness (p38/MPAK)	ING4	Ketr-3 (renal carcinoma), 786-O (renal carcinoma)	Overexpression accelerated xenograft growth and lung metastasis and raised sphere formation and stem markers (OCT4/CD44/NANOG/c-MYC); knockdown lowered both; ING4 repressed DUSP4, activating p38 MAPK and type I ISGs, with p38 inhibition and DUSP4 rescue confirming the axis (pro-malignant)	Overexpression + knockdown (cell line) + xenograft (*in vivo*, OE only) + TCGA correlation	Tang et al. (2022)
Differentiation (c-MYC-induced)	ING4	iPrECs (immortalized prostate epithelial cells), (prostate) +	c-MYC-driven transient ING4 required for basal-to-luminal differentiation; shRNA knockdown blocked differentiation and, with c-MYC/ERG, produced orthotopic tumors (60% vs. 0%); PHD-deletion mutant failed to drive differentiation, implicating chromatin reading	Knockdown + overexpression + domain mutant (cell line) + orthotopic xenograft (*in vivo*)	Berger et al. (2014)
PI3K/AKT	ING5	A549 (lung)	Knockdown activated PI3K/AKT and IL-6/STAT3 (reversed by overexpression); PI3K/STAT3 inhibition reversed the knockdown phenotype and reduced metastasis (tumor-suppressive)	Knockdown + overexpression (cell line) + xenograft (*in vivo*)	Liu et al. (2017)
PI3K/AKT	ING5	TPC1 (thyroid)	Overexpression blunted HGF-c-Met/PI3K/AKT, suppressed invasion/EMT, and reduced growth and lung metastasis (tumor-suppressive)	Overexpression (cell line) + xenograft (*in vivo*)	Gao and Han (2018)
PI3K/AKT	ING5	PC3, LNCaP (prostate)	Overexpression decreased p-AKT and malignant potential; p53-independent in PC3 (tumor-suppressive)	Overexpression (cell line)	Barlak et al. (2019)
PI3K/AKT	ING5	BT189 (glioblastoma BTIC)	Overexpression promoted self-renewal/stemness, upregulated CACNA1D/TRPM3/FSHR/FSHB (ChIP), and activated PI3K/AKT and MEK/ERK; PHD-dependent (pro-malignant)	Overexpression + ChIP (cell line)	Wang et al. (2018)
EMT (miR-34c-5p/TGF-β)	ING5	A549, H1299 (lung)	Overexpression induced miR-34c-5p, repressing Snail1 and TGF-β/Smad3; knockdown promoted EMT/invasion and metastasis; miR-34c-5p restoration reversed (tumor-suppressive)	Overexpression + knockdown (cell line) + xenograft (*in vivo*)	Yang et al. (2023)
Glycolysis TIE1/PDK1	ING5	A549, H1299 (ling), HCT116 (colorectal) + tissue	Overexpression upregulated TIE1, phosphorylating PDK1-Y163 and lowering glycolysis; PDK1-Y163F abolished effects; low p-PDK1-Y163 linked to poorer survival (tumor-suppressive, metabolic)	Overexpression + knockdown (cell line) + xenograft (*in vivo*) + clinical	Zhang et al. (2024)
Nuclear-cytoplasmic shift	ING5	Colorectal carcinoma (patient tissue)	Progressive nuclear-to-cytoplasmic shift; total protein elevated, mRNA unchanged (relocalization); nuclear ING5 inversely and cytoplasmic positively associated with tumor size, invasion depth, and dedifferentiation; not prognostic (tumor-suppressive via nuclear loss)	Clinical (IHC + western)	Zheng et al. (2011)
INCA1 axis	ING5	AML blasts (clinical) + mouse bone marrow/fibroblasts, COS-7 (African green monkey kidney)	mRNA reduced in AML; ING5-INCA1 interaction; overexpression suppressed colony formation, proliferation, and S-phase and raised anti-Fas apoptosis, all INCA1-dependent; overexpression-only in mouse (tumor-suppressive, unconfirmed in human)	Overexpression (mouse cells) + clinical mRNA	Zhang et al. (2011)
Proliferation/survival	ING5	HCT116 (colorectal), U2OS (bone), HeLa (cervix)	shRNA depletion suppressed proliferation and induced caspase-dependent, p53-independent apoptosis; overexpression only minor (counter to tumor suppression; ING5 required for proliferation/survival)	Knockdown + overexpression (cell line)	Linzen et al. (2015)

### 4.1 Activation of p53-pathway

The most frequently altered gene in human cancers is *p53*, with mutations occurring in the majority of malignancies. Activation of p53 is triggered by damaged DNA or other forms of cellular stress, leading to cell cycle arrest, senescence, or apoptosis (Wang et al., 2026). The interaction between ING4/5-p300 and ING5-TIP60 (Section 2.3) suggests that both ING proteins serve as p53 co-factors for tumour suppression (Shiseki et al., 2003; Liu et al., 2013; Zhang et al., 2017). Additionally, both ING4 and ING5 were found to physically interact with p53 in RKO cells, as determined by co-immunoprecipitation (Shiseki et al., 2003). However, paralog-specific roles are not identical. ING4 exhibited more potent promotion of acetylation of p53 at K382 compared to ING5. On the other hand, in the same study overexpression of ING5 in RKO cells led to stronger P21 activation, a transcriptional target of p53 (Shiseki et al., 2003). Moreover, unlike ING5, ING4 has not yet been found to activate p53 by interacting with TIP60 (Liu et al., 2013). This pattern is consistent with ING4 and ING5 directing p53 toward partially distinct outputs rather than acting in an interchangeable manner. In prostate cancer, ING5 was found to be downregulated in both tissue samples and cell lines compared to the controls. Its overexpression in LNCaP cells that are wild-type for *p53* increased p53 and its acetylated forms (K120 and K373/382), though this observation rested on correlative overexpression (Barlak et al., 2019). The same study also found that ING5 overexpression suppressed proliferation, clonogenicity, migration, and invasion in the p53-null PC3 line, indicating that its anti-tumor activity is not solely p53-dependent. The knockdown of ING4 in LNCaP cells led to differential expression of genes related to cancer, organismal injury, and abnormalities as determined by RNA-seq analysis, with changes in expression of key genes validated by RT-qPCR (Shatnawi et al., 2020). However, whether ING4 has the potential to inhibit the malignancy of prostate cancer remains unknown.

The strength of evidence supporting the roles of ING4/5 in activating the p53 pathway is uneven. Links of ING4 and ING5 to the p300 axis rest primarily on ectopic overexpression of ING4 or ING5 in RKO (Shiseki et al., 2003) and A549 cells (Zhang et al., 2017). The TIP60 axis is supported with loss-of-function evidence: shRNA knockdown of endogenous ING5 in U2OS cells reduced p53K120 acetylation, *BAX* and *GADD45* induction, and suppressed doxorubicin-induced apoptosis (Liu et al., 2013). Nevertheless, direct *in vivo* confirmation that loss of endogenous ING4 or ING5 phenocopies p53-pathway disruption in mammalian tissues remains lacking, which may be unsurprising given the breadth of p53 target genes.

Indirect evidence for the functional importance of this axis comes from virus biology. The Epstein-Barr virus (EBV)-encoded EBNA3C protein binds both ING4 and ING5 in Saos-2 (p53-null) cells, occupying the same regions of ING4 (residues 66–184) and ING5 (residues 120–240) that engage p53, and competitively displaces p53 from both paralogs in a dose-dependent manner in *p53* ^−/−^ *Mdm2* ^−/−^ MEFs. Interestingly, ING4 was found to bind both p53 and EBNA3C via its NLS1 domain, whereas ING5 bound them via its PHD finger (Saha et al., 2011). EBNA3C represses ING4-and ING5-enhanced p53 transactivation of the p21 promoter, suppresses ING-mediated p53-dependent apoptosis, and rescues colony formation in p53-transfected Saos-2 cells. The endogenous EBNA3C-ING4 and EBNA3C-ING5 complexes are detectable in EBV-transformed lymphoblastoid cell lines (Saha et al., 2011). These results indicate that suppression of p53 by competitive binding with ING4/5 via distinct domains could be one of the mechanisms by which EBNA3C promotes proliferation.

### 4.2 NF-κB pathway

Repression of NF-κB-dependent transcription is one of the best-characterized tumor-suppressive functions of ING4 and has also been reported for ING5 in gastric cancer models, but with varied, and possibly cell type-specific results (Gou et al., 2015; Zhang et al., 2018; Du et al., 2019; Shatnawi et al., 2021a). The NF-κB transcription factors are activated in diverse types of human cancers. NF-κB regulates genes encoding proteins implicated in cell proliferation, survival, inflammation, invasion, metastasis, and tumor drug resistance (Xia et al., 2014). ING4 interacts with p65 (RELA), a core component of NF-κB, to inhibit transcription of its target genes (Garkavtsev et al., 2004; Nozell et al., 2008) and can also activate the IκB promoter to suppress NF-κB signaling and innate immunity (Coles et al., 2010). In the glioblastoma cell line U87MG, the repression of ING4 induced an increase in the binding of NF-κB to DNA and increased the expression of NF-κB-regulated genes (IL-6, IL-8, COX-2, CSF3). The overexpression of ING4, on the other hand, reduced growth rates in control and antisense-ING4 tumors (Garkavtsev et al., 2004). Further experiments in glioma cells null for ING4 (U251-MG) or silenced for ING4 (U118-MG) established that ING4 distinctly inhibits NF-κB signaling; namely, ING4 does not block p65 binding to DNA as was initially suggested (Garkavtsev et al., 2004), but binds to p65 already bound to the promoter, dephosphorylates p65 at Ser536, displacing p300, recruiting HDAC-1, and lowering H3 and H4 acetylation and H3K4me3 at NF-κB target promoters (Nozell et al., 2008). An alternative mechanism by which ING4 inhibits p65 was proposed by Hou et al. (2014) using *in vitro* reconstitution with recombinant proteins: the ING4 PHD finger acts as an E3 ubiquitin ligase, partnering with the E2 enzyme UbcH3 to catalyze K48-linked polyubiquitination of p65 at K62, thereby targeting it for proteasomal degradation. The activity requires the ING4 C239 residue, and cell-based assays confirm that it is NLS-independent and functions in both the cytoplasm and the nucleus (Hou et al., 2014). Notably, Nozell et al. (2008) reported that p65 occupancy at the *COX-2* and *MMP-9* promoters was unchanged by ING4 induction, an observation difficult to reconcile with the substantial p65 degradation observed in their system. Similarly, another study observed no change in p65 protein levels with either ING4 overexpression or knockdown in mouse macrophages (Yang et al., 2020). Thus, ING4 may inhibit p65 activity through multiple context-dependent mechanisms. ING4-mediated NF-κB suppression has been observed in astrocytoma (Klironomos et al., 2010), melanoma (Li and Li, 2010), breast (Byron et al., 2012; Yan et al., 2015), gastric (Li et al., 2013; Chen et al., 2018b), and osteosarcoma (Li et al., 2014), with consistent effects on downstream targets. The factors and cofactors responsible for triggering specific ING4 mechanisms clearly require further investigation.

ING5 effects on NF-κB are tissue-specific, and the mechanisms involved are less firmly established than for ING4. In esophageal squamous cell carcinoma, ING5 overexpression reduced total levels of p65, a potent NF-kB activator, p-AKT (Kim et al., 2006) and MMP-9, and reduced cancer progression (Zhang et al., 2018). On the other hand, in SGC-7901 gastric cells, ING5 overexpression increased NF-κB protein levels and promoter activity, cytokine transcription (IL-1, IL-2, IL-4, IL-10, IL-17), and conferred chemoresistance. However, the same overexpression also suppressed proliferation, metastasis, migration, and invasion (Gou et al., 2015).

The studies of ING4 in gastric cancer models demonstrated that ING4 suppressed NF-κB in MKN-45 cells and MNNG-transformed gastric epithelial cells (Li et al., 2013; Chen et al., 2018b), establishing potential paralog-specific divergence within gastric cancer. However, results of both studies of ING5 effects on NF-κB rest solely on overexpression. In contrast, Gou et al. (2015) performed ING5 siRNA knockdown in GT-3TKB cells, but only for proliferation, cell-cycle, apoptosis, and invasion phenotypes, not for NF-κB regulation. These studies used indirect readouts such as NF-κB protein levels estimated by Western blotting (Gou et al., 2015) without chromatin-level analyses comparable to glioma ING4 work (Nozell et al., 2008) and disagree on the direction of AKT regulation by ING5 (p-Akt decreased in Zhang et al., 2018 vs. p-Akt increased in Gou et al., 2015). The mechanistic basis for this discrepancy remains undefined, but two non-exclusive candidates emerge from the existing data. First, the two studies may not assay the same NF-κB species: Zhang et al. (2018) measured p65, whereas Gou et al. (2015) did not specify the subunit detected by Western blot and identified only the non-canonical p100 (NFKB2) subunit among the upregulated proteins in their proteomic screen. This finding suggests that the effects of ING5 on NF-kB in gastric tissues are likely mediated through the alternative pathway, rather than the canonical pathway involving RELA, which ING4 modulates. Second, unlike ING4, ING5 interacts with MOZ/MORF enzymes in addition to HBO1, so tissue-specific differences in the availability of KAT partners may affect transcriptional activity. Resolving this will require subunit-resolved, endogenous loss-of-function analyses in matched gastric and esophageal models.

### 4.3 Wnt/β-catenin pathway

Suppression of canonical Wnt/β-catenin signalling has been reported for both paralogs, with mechanistic resolution being stronger for ING5. ING5 promotes β-catenin degradation via phosphorylation-dependent mechanisms in lung cancer cell lines. ShRNA silencing of ING5 increased total β-catenin in A549 lung adenocarcinoma and H1299 non-small cell lung carcinoma cell lines, and decreased Ser33/37 (GSK-3β kinase target site) phosphorylation in A549, with β-catenin mRNA unchanged in A549. ING5 overexpression reduced total β-catenin levels in A549 and H1299 cells, accompanied by increased phosphorylation at Ser33/37. The Wnt target c-MYC decreased with ING5 overexpression. However, the effect was more pronounced in the colorectal cancer cell line HCT116 than in A549 or H1299 (Liu et al., 2019). Tankyrase inhibition with XAV939, which stabilizes Axin and promotes β-catenin degradation, restored Ser33/37 phosphorylation and attenuated the proliferation, migration, invasion, and epithelial to mesenchymal transition (EMT) phenotypes of ING5 knockdown in A549 (Liu et al., 2019). The upstream step by which ING5 engages GSK-3β-mediated phosphorylation is, however, undefined.

Mechanistic evidence for ING4 in Wnt suppression is limited to total protein-level observations in A549. Li et al. (2008a) showed western blots indicating decreased Wnt-1 and cyclin D1 (a Wnt/β-catenin target), with a seemingly slight decrease in β-catenin, in A549 cells overexpressing ING4. These changes accompanied reduced colony formation and smaller xenografts in the same ING4-overexpressing cells. However, no quantification of the Western blot results was performed. Subsequently, Tang et al. (2019) showed that miR-650 directly targets and represses ING4. In A549 cells, miR-650-driven changes in ING4 were accompanied by opposite changes in Wnt-1 and β-catenin levels: miR-650 inhibition increased endogenous ING4 and reduced Wnt-1 and β-catenin; a miR-650 mimic produced the opposite effect. ING4 re-expression in mimic-treated cells partially reversed the increase measuring phospho-β-catenin, β-catenin nuclear localization, TCF/LEF reporter activity, and a broader Wnt target panel would add to a better mechanistic understanding of the role of ING4 in regulating this pathway.

Interestingly, upregulation of HBO1 was found to promote accumulation of nuclear β-catenin, TCF/LEF activity, and Wnt targets (c-MYC, c-JUN, CCND1, CTLA4, LEF1, TCF1, and Axin2) in J82 and T24 bladder carcinoma, with bidirectional XAV939 and LiCl rescue confirming Wnt-dependence (Chen et al., 2018a). It is not clear if the effects of the ING4/5-HBO1 complex on the Wnt/β-catenin pathway are opposite to those of the free HBO1 or whether the differences in observations between lung versus bladder carcinoma cell lines are tissue-specific. All evidence of ING4/5 effects on Wnt/β-catenin comes from cancer cell lines and spans both overexpression and loss of function, with the endogenous support limited to two lung cancer cell lines: ING5 shRNA in A549 and H1299 (Liu et al., 2019) and miR-650 modulation of endogenous ING4 in A549 (Tang et al., 2019). Whether endogenous ING4/5 regulates Wnt in other tissues or cell types remains untested.

### 4.4 Autophagy

The best-controlled experiments generating data linking ING4 and ING5 to autophagy indicate that endogenous ING4 acts as a tonic repressor of the BECN1/PIK3C3 autophagy initiation complex (Sica et al., 2017). In contrast, data indicating pro-autophagic roles are derived solely from supraphysiological overexpression (Gong et al., 2013). For ING5, no loss-of-function data are available, and overexpression studies gave variable results (Gou et al., 2015).

Depletion of ING4 by three non-overlapping siRNAs raised autophagic flux in both U2OS osteosarcoma and HCT116 colorectal carcinoma cells, shown by GFP-LC3B puncta persisting under bafilomycin A1, and in U2OS by raised LC3B (MAP1LC3B) lipidation and lowered SQSTM1 levels. In U2OS, the increase required BECN1 and PIK3C3. It was accompanied by elevated PIK3C3 activity, and co-immunoprecipitation showed an ING4/BECN1 association in both lines. This was retained after p300/p53 loss in HCT116 but lost on starvation in U2OS, indicating that ING4 represses autophagy, likely by restraining the lipid-kinase activity of the BECN1/PIK3C3 complex. Overexpression of ING4 did not alter basal or induced autophagy in either cell line (Sica et al., 2017).

By contrast, adenoviral ING4 overexpression in U251MG and LN229 glioma cells triggers death with autophagic and apoptotic features, with partial rescue by 3-methyladenine or by BECN1 or ATG7 knockdown, suggesting that autophagy is a causal death mechanism parallel to apoptosis (Gong et al., 2013). This observation does not necessarily conflict with the repressive role of endogenous ING4 noted by Sica et al. (2017). The ING4-induced autophagy in U251MG and LN229 was a component of overexpression-induced death (Gong et al., 2013) and thus, could indicate a cytotoxic feature of the cell death program rather than indicating that ING4 positively regulates the autophagy pathway.

For ING5, the evidence for autophagy regulation is very limited, resting on a single overexpression study in the SGC-7901 gastric cancer cell line (Gou et al., 2015). Ectopic ING5 was reported to increase autophagy, but its two morphological readouts, LC3B-EGFP puncta and autophagosomes detected by electron microscopy, appear only as unquantified representative images. The rise in BECN1 protein following ING5 overexpression is a weak marker on its own and was accompanied by reduced apoptosis and increased resistance to six cytotoxic agents, a pro-survival rather than tumor-suppressive phenotype. On the same blots, however, ATG7 and ATG14, the proteins that build autophagosomes and LC3 puncta, both decreased, while the autophagy-suppressing PI3K, phospho-AKT, and p70S6K rose. These discordances were not addressed by the authors (Gou et al., 2015). In the absence of flux assays, loss-of-function assays and some discordant observations, whether ING5 regulates autophagy in cancer models remains unresolved.

### 4.5 Paralog-specific roles in cancer

All members of the ING family of epigenetic regulators, particularly the first ones examined in detail have been implicated in affecting cancers of various types (Gong et al., 2005; Heliez et al., 2023), and more recently both ING4 and ING5 have been implicated in numerous forms.

#### 4.5.1 ING4-specific roles

ING4 appears to restrain cell motility through cytoplasmic interactions. In RKO colon carcinoma cells, mass spectrometry of ING4 pull-downs identified the focal-adhesion scaffold liprin α1 (PPFIA1) as a binding partner that was confirmed by co-immunoprecipitation, mapped to ING4 residues 135–180, and was absent in ING2 and ING5. Cytoplasmic ING4 colocalized with liprin α1 at lamellipodia near vinculin and was also seen in HEK-293 and U-87 MG glioblastoma cells. ING4 overexpression suppressed cell spreading and migration, whereas four independent siRNAs against endogenous ING4 increased both. ING4 lost its suppressive effect when liprin α1 was knocked down (Shen et al., 2007).

ING4 also represses c-MYC translation. Through its NLS region, ING4 binds AUF1. ING4, AUF1, and c-MYC mRNA occupy a single ribonucleoprotein complex on the c-MYC 3′UTR. ING4 overexpression lowers, and knockdown raises c-MYC protein levels in both K562 and HeLa cells, whereas c-MYC mRNA (shown in K562) and c-MYC protein turnover (shown in HeLa) are unchanged, placing the effect at the level of translation (Lu et al., 2013a).

In multiple myeloma cell lines, ING4 associates with HIF prolyl hydroxylase 2 (PHD2/EGLN1). Under hypoxic conditions, ING4 knockdown raised HIF-1α DNA-binding activity, leading to an increase in the level of its target gene encoding NIP-3, while HIF-1α level remained stable (Ozer and Bruick, 2005). Additionally, there was an increase in HIF-1α-dependent, IL-8- and osteopontin-driven angiogenesis. However, ING4 overexpression alone was not sufficient to reduce IL-8 or osteopontin levels in these studies (Colla et al., 2007).

In melanoma, ING4 transcriptionally activated the ARF like GTPase six interacting protein 5 (JWA), which, in turn, lowered the level of integrin linked kinase (ILK) and dampened ILK/IL-6/VEGF-induced angiogenesis. Across a tissue microarray, JWA fell, and ILK rose from dysplastic nevus to metastasis, with low JWA and high ILK each predicting poorer survival (Lu et al., 2013b).

By contrast, in renal cell carcinoma ING4 showed pro-malignant activity. ING4 overexpression in 786-O cells accelerated subcutaneous xenograft growth and increased the EMT number of lung metastatic nodules in a tail-vein model. The same overexpression raised sphere formation and the stem-cell markers OCT4, CD44, NANOG, and c-MYC in Ketr-3 and 786-O cells while ING4 knockdown reduced both. ING4 repressed the p38 phosphatase DUSP4, activating the p38 MAPK signalling pathway and inducing the type I interferon-stimulated genes IFITM1, MX2, and OAS2. A p38 MAPK inhibitor abolished sphere formation and ING4-driven overexpression of IFITM1, MX2, and OAS2. On the other hand, DUSP4 silencing restored p38 activity and sphere formation in ING4-knockdown cells. ING4 and DUSP4 mRNA expression were inversely correlated across the TCGA KIRC and KIRP cohorts. The *in vivo* phenotype depends solely on overexpression (Tang et al., 2022). Taken together, the results of this study indicate that ING4 may exert its pro-tumorigenic effects in RCC by maintaining stemness.

In immortalized human prostate epithelial cells transient ING4 expression was found to suppress tumorigenesis by promoting basal-to-luminal differentiation, a transition read out by loss of integrin α6 and gain of the androgen receptor (AR). c-MYC drove ING4 expression during this transition, and ING4 acted downstream of c-MYC: c-MYC knockdown abolished ING4 induction and blocked differentiation, which was rescued by ING4 re-expression. The shRNA knockdown of ING4 blocked differentiation and, together with c-MYC and ERG overexpression, produced orthotopic tumors in 60% of mice, versus none when c-MYC and ERG were overexpressed without the loss of ING4. A C-terminal ING4 mutant lacking the PHD finger that binds H3K4me3 did not promote differentiation, suggesting that ING4 must read chromatin to drive differentiation. Using a tissue microarray, the authors also found that ING4 was downregulated in 32/50 (>60%) of the surveyed prostate cancer tumors, supporting its role as a tumour suppressor in this malignancy (Berger et al., 2014).

#### 4.5.2 ING5-specific roles

For ING5, paralog-specific findings mostly center on PI3K/AKT where its effects were found to be both tumor-suppressive (Liu et al., 2017; Gao and Han, 2018) and pro-malignant (Wang et al., 2018). ING5 appeared to restrain EMT, mediated through the PI3K/AKT and IL-6/STAT3 pathways within epithelial carcinoma cell lines. In A549 cells, ING5 knockdown activated the PI3K/AKT and IL-6/STAT3 pathways, which was reversed by ING5 overexpression. The inhibition of PI3K or STAT3 reversed the ING5 knockdown phenotype *in vitro* and reduced tail-vein metastasis (Liu et al., 2017). In thyroid cancer cells, ING5 overexpression in the TPC1 line blunted hepatocyte growth factor (HGF) driven c-Met/PI3K/AKT signalling, suppressed HGF-induced invasion and EMT *in vitro*, and reduced tumor growth and lung metastasis in xenografts (Gao and Han, 2018). Additionally, overexpression of ING5 in prostate cancer cell lines PC3 and LNCaP decreased p-AKT levels and reduced malignant potential. Since PC3 is p53-null, ING5 appears to exert its tumor-suppressive properties in a p53-independent manner in this cell line, potentially by suppressing AKT activity (Barlak et al., 2019).

However, in contrast to these effects, possible pro-malignant activity of ING5 was observed in glioblastoma brain tumour initiating cells (BTICs), implicating PI3K/AKT pathway activation (Wang et al., 2018). In BT189 BTICs, ING5 overexpression promoted self-renewal and cancer stem-like properties. The overexpressed ING5 upregulated calcium-channel (*CACNA1D, TRPM3*) and FSH-pathway (*FSHR, FSHB*) genes, with ChIP confirming endogenous occupancy. These effects, in turn, induced calcium channel activity and activated the PI3K/AKT and MEK/ERK pathways, increasing the stemness of BTICs. A PHD-deletion mutant abolished the effects supporting a nuclear role. The negative regulation of PI3K/AKT by ING5 in the A549 (lung) (Liu et al., 2017) TPC1 (thyroid) (Gao and Han, 2018), and potentially PC3 (prostate) (Barlak et al., 2019) carcinoma lines and its positive regulation in BT189 BTICs (Wang et al., 2018) are not strictly contradictory, since the carcinoma lines are bulk tumor cells and BT189 is stem-like. None of the studies showed ING5 acting directly on the pathway, and all relied on ectopic perturbation. Although two of the carcinoma studies extended to xenograft models, these experiments investigated metastasis and tumor growth rather than PI3K/AKT activity (Liu et al., 2017; Gao and Han, 2018). Therefore, the conditions under which ING5 restrains versus reinforces PI3K/AKT *in vivo* remains unresolved. Interestingly, the pro-malignant activity of ING5 in BTICs (Wang et al., 2018) parallels reports of pro-malignant activity of ING4 in RCC (Tang et al., 2022): both exert pro-oncogenic effects by restraining differentiation of the respective cell lines, albeit via activating different pathways: p38/MPAK for ING4 (Tang et al., 2022), and PI3/AKT and MEK/ERK for ING5 (Wang et al., 2018). Thus, future studies should investigate the activity of ING4 and ING5 in cancer stem cells (CSCs) from other cancers asking whether they promote stemness and if their functions are interchangeable. If confirmed, both paralogs could be potential therapeutic targets in CSCs.

Several other potential ING5-dependent tumor-suppression mechanisms have been identified: (i) ING5 overexpression induced miR-34c-5p, which repressed its target SNAIL1 and the downstream TGF-β/SMAD3 signalling pathway. ING5 knockdown in A549 and H1299 promoted EMT, proliferation, migration, and invasion. The loss of ING5 in A549 also increased metastasis in a tail-vein xenograft model. Restoring miR-34c-5p reversed all these effects (Yang et al., 2023). (ii) In another study, ING5 overexpression in A549 and H1299 lung cancer cells upregulated the orphan kinase TIE1, which phosphorylated a glycolytic enzyme, pyruvate dehydrogenase kinase 1 (PDK1), at a new inhibitory site, Y163, decreasing glycolysis and increasing oxidative phosphorylation, an effect reproduced in HCT116 colorectal cells. The p-PDK1 Y163 mark decreased with ING5 knockdown. A phospho-dead PDK1 Y163F mutant abolished ING5’s metabolic and tumor-suppressive effects both *in vitro* and in A549 xenografts. A lower p-PDK1 Y163 in lung adenocarcinoma tissue from cancer patients was associated with poorer overall survival (Zhang et al., 2024). (iii) In colorectal carcinoma, ING5 was found to undergo a progressive nuclear-to-cytoplasmic shift, with nuclear positivity falling from normal mucosa through adenoma to carcinoma. Cytoplasmic ING5 was detected in 85% of carcinomas, while total protein was elevated and mRNA unchanged, indicating relocalization rather than loss. Nuclear ING5 was inversely associated with tumor size, invasion depth, and degree of dedifferentiation. On the other hand, the cytoplasmic ING5 was positively associated with them, although localization did not predict survival (Zheng et al., 2011).

Zhang et al. (2011) reported that ING5 mRNA is reduced in AML blasts relative to normal bone marrow, and that ING5 associates with the inhibitor of CDK, cyclin A1 interacting protein 1 (INCA1) *in vitro* and upon overexpression, and *in vivo* in the COS-7 African green monkey immortalized fibroblast-like kidney cell line. In mouse cells, ING5 overexpression reduced colony formation in primary bone marrow and, in fibroblasts, suppressed proliferation and S-phase progression and increased sensitivity to anti-Fas antibody-induced apoptosis. All these effects required Inca1 and were lost in *Inca1* ^*−/−*^ cells, where ING5 overexpression instead mildly promoted proliferation under conditions of serum starvation. However, these functional data rely on ectopic overexpression in mouse cells, and Inca1 was never assayed in the AML samples (Zhang et al., 2011). Therefore, further studies in human leukemia are needed to investigate the potential role of the ING5/INCA1 axis in AML.

Contrary to the tumor-suppressive role of ING5, another study found that shRNA depletion of endogenous ING5 strongly suppressed proliferation in HCT116, U2OS, and HeLa cells and induced caspase-dependent, p53-independent apoptosis in HCT116, whereas overexpression of ING5 produced only minor effects (Linzen et al., 2015). The authors describe the prior tumor-suppressor evidence as largely correlative and regard the requirement they observe as consistent with a previously reported MCM-associated role for ING5 in DNA replication (Doyon et al., 2006). This replication role was not assayed in their study, so the basis of the discrepancy with the overexpression literature remains open.

### 4.6 Potential translational and clinical implications

Downregulation and mislocalization of ING4/5 have prompted interest in their potential as clinical biomarkers. The prognostic value of ING4 and ING5 has been examined in several tumor types, but the direction and robustness of these associations are neither uniform nor consistent. For ING4, reduced expression has most often been associated with worse outcomes: low ING4 predicted poorer survival in primary melanoma (Li et al., 2008b), gastrointestinal stromal tumors (Nanding et al., 2014), and colorectal cancer (Chen et al., 2016). High ING4 mRNA levels were associated with longer overall survival in a large breast cancer dataset. However, the effect was weak and largely confined to premenopausal and non-luminal subgroups (Shatnawi et al., 2021b). This direction is, however, not universal, as ING4 was found to be pro-malignant in RCC (Tang et al., 2022). For ING5, the prognostic signal is less consistent and depends on subcellular localization. Higher nuclear ING5 protein levels predicted better overall survival in gastric (Xing et al., 2011) and lung (Zhang et al., 2015) cancers. In contrast, its higher cytoplasmic localization was associated with poorer survival in colorectal carcinoma (Zheng et al., 2011). In gastric cancer, however, the association between higher nuclear ING5 levels and better survival lost significance after adjusting for tumor depth, UICC stage, and Lauren’s classification (Xing et al., 2011). When ING5 mRNA levels were measured, the direction was not consistent across cancers: higher mRNA was associated with better relapse- and metastasis-free survival in breast cancer (Ding et al., 2017) but shorter progression-free survival in ovarian and gastric cancers (Zheng et al., 2017; Zheng et al., 2022b) and lower overall survival in gastric cancer (Zheng et al., 2022b). Taken together, ING4 and ING5 carry prognostic information in several cancers, but what is informative (the paralog, the direction, the subcellular localization, and whether protein or transcript is measured) differs by the malignancy type, so any biomarker application would need to be defined in a cancer type- and subcellular-specific manner.

Because of their tumor-suppressive activities, ING4 and ING5 may potentially be re-expressed in tumors as a therapeutic strategy. Ectopic ING4 expression or gene delivery reduced tumor growth and angiogenesis in a glioblastoma xenograft model (Garkavtsev et al., 2004), suppressed proliferation and xenograft growth in lung adenocarcinoma (Li et al., 2008a), and inhibited proliferation and invasion while inducing apoptosis in osteosarcoma cells (Li et al., 2014). In a lung cancer xenograft model, overexpression of ING5 suppressed tumor growth through apoptosis and inhibition of proliferation (Zhao et al., 2016). An alternative possibility is to target ING4/5-dependent stemness. Both paralogs were observed to promote self-renewal in cancer models: ING4 in RCC (Tang et al., 2022) and ING5 in BTICs (Wang et al., 2018). However, both strategies carry the same caveat. Because ING4 and ING5 appear to be pro-malignant in stem-like cancer cells yet tumor-suppressive in more differentiated models, indiscriminate overexpression or suppression is unlikely to be uniformly beneficial across cell types and could even be harmful. Tumor type and differentiation state should therefore be considered when choosing between re-expression and inhibition strategies for targeting ING4/5.

**FIGURE 5:**
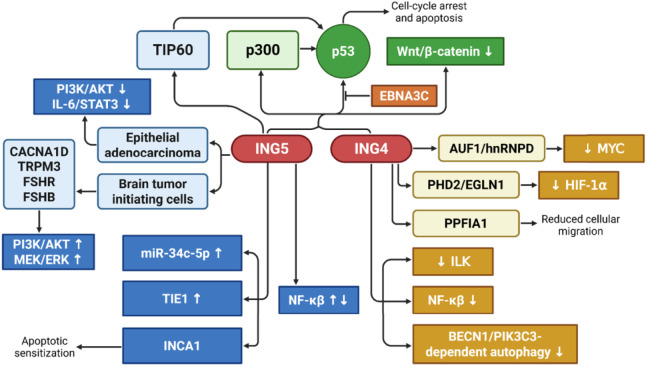
ING4 and -5 in cancer. Both ING4 and ING5 have been linked to some overlapping and various independent functions in cancer models. Evidence suggests ING4 and -5 associate with p300, driving p53 acetylation and increases in cell-cycle arrest and apoptosis (Liu et al., 2013; Zhang et al., 2017). Each can physically bind p53, a process that can be disrupted by EBNA3C, encoded by the Epstein-Barr virus (EBV) (Shiseki et al., 2003; Saha et al., 2011). Both proteins have been linked to a decrease in Wnt/*β*-catenin activity (Liu et al., 2019). Each protein has been linked to tumor suppressive pathways, an observation more consistent across ING4 studies. Some studies have proposed intermediaries, such as AUF1, PHD2 and PPFIA1, which drive down the expression of proteins associated with tumor progression or appear to diminish cancer cell aggression (Shen et al., 2007; Colla et al., 2007; Lu et al., 2013a). Additionally, ING4 expression appeared to decrease NF-κ*β* and autophagy (Garkavtsev et al., 2004; Nozell et al., 2008 Sica et al., 2017). In contrast, ING5’s role in NF-κ*β* repression varied across tissues, acting as a promoter of cellular proliferation and survival in certain cell lines (Gou et al., 2015; Zhang et al., 2018). Independent tumor suppressive associations of ING5 include its link to TIP60 that acetylates p53 to drive pro-apoptotic pathways (Liu et al., 2013). Furthermore, ING5 expression has been linked with other independent factors that promote apoptosis or sensitize malignant cells to increased DNA damage and destruction (Zhang et al., 2011; Yang et al., 2023; Zhang et al., 2024). Another contrast in ING5’s role in tumor suppression is in its links to PI3K/AKT and IL-6/STAT3 or MEK/ERK pathways. In certain tumor cell lines, specifically epithelial adenocarcinoma, ING5 overexpression was linked to decreases in pro-tumor PI3K/AKT and IL-6 pathways (Liu et al., 2017; Barlak et al., 2019). However, in glioblastoma associated stem cells, ING5’s pro-proliferative role was linked to an increase in PI3K/AKT and MEK/ERK activity, potentially mediated through calcium signaling pathways (Wang et al., 2018).

A schematic of the major pathways affected by ING4 and ING5 in cancers is shown in Figure 5.

## 5 ING4 and ING5 in differentiation and development

The roles of ING4 and ING5 extend beyond cancer. Both proteins, together with their acetyltransferase complexes MOZ/MORF and HBO1, have been linked to differentiation and development. Their roles in development have only recently begun to be explored. This section follows that work across embryonic and adult stem cells and in tissue and organ formation in mice and flies. The summary of the findings presented in this section along with the evidence supporting them are presented in Table 2.

**TABLE 2:** Roles of ING4 and ING5 in differentiation and development.

System/Model (species)	ING and perturbation	Key finding	Mechanism: complex/Mark/Pathway	Paralog relationship	References
Embryonic stem cells - mouse (mESC lines), human (ESC→trophoblast)	ING5: differentiation time-course; mESC *Ing5* KO; knockdown	ING5 falls as cells differentiate; *Ing5*-KO embryoid bodies ∼27% larger, pluripotency markers (*Sox2*, *Nanog*, *Oct4*, *Klf4*) drop but do not drive differentiation alone → ING5 restrains differentiation	ING5 targets SET1A (H3K4 methyltransferase) to development/morphogenesis genes, not required for SET1A recruitment; loss lowers H3K23ac (H3K14ac by mass-spec only); H4 acetylation unchanged	ING4 not studied	Wang et al. (2018), Cenik et al. (2022)
Epidermal stem cells/keratinocytes - human	ING5: siRNA/shRNA knockdown	ING5 sits in a self-renewal network (SMARCA5, BPTF, EZH2, UHRF1); depletion cuts clonogenicity and promotes differentiation → maintains undifferentiated state	ING5 at H3K4me3 active promoters (*ITGB1* > *ITGA6*); ZMAT2-regulated splicing enriched at ING5 sites	ING4 not studied	Mulder et al. (2012), Tanis et al. (2018)
Neural stem cells, SVZ - mouse	*Ing5* germline KO	↓Sox2, ↓nestin, ↑doublecortin-positive new neurons → supports NSC maintenance	Likely via MOZ/MORF (MORF an NSC marker; MOZ self-renewal via p16^INK4a^) but untested	ING4 not studied	Al Shueili et al. (2025) (MOZ/MORF: Sheikh et al. (2012), Perez-Campo et al. (2014))
Mesenchymal stem cells - human	ING5: siRNA knockdown; miR-193 overexpression (lowers ING5)	ING5 knockdown ↑proliferation, ↑CDK2/PCNA → restrains MSC proliferation; ING5 a direct miR-193 target	Post-transcriptional miR-193 targets ING5 inhibiting it	ING4 not studied	Wang et al. (2012)
Mouse embryo - whole animal	*Ing4* KO; *Ing5* KO; *Ing4;Ing5* double KO (germline)	*Ing4* ^−/−^ viable, normal (LPS-sensitive, ↑NF-κB); *Ing5* ^−/−^ viable but abnormal (subfertile, small litters; perinatal loss; blood defects; DLBCL with age); double KO lethal, arrest at E8.5	Double KO phenocopies HBO1 loss (E8.5 arrest)	Redundant for embryonic survival (single KOs survive longer)	Coles et al. (2010), Mah et al. (2023), Al Shueili et al. (2025), Mah et al. (2024)
Mouse embryo - *Hox* loci/body segmentation	*Moz* KO (ING5 occupancy readout); *Ing5* loss-of-function not tested	*Moz* KO → ↓ING5 at *Hoxa*/*Hoxb*, locus-specific H3K9 hypoacetylation, ↓Hox expression, segment defects; retinoic acid rescues H3K9ac and segments but not ING5 presence at the *Hoxa/b* loci	MOZ complex; H3K9ac at *Hox*	Whether ING5 itself is required - untested (open question)	Voss et al. (2009)
Heart - mouse (embryonic allelic series; postnatal)	Postnatal ING5; germline *Ing4;Ing5* allelic series	Postnatal: ING5 restrains cardiomyocyte proliferation (p21; LRP6-stabilized mRNA). Embryonic: *Ing5* ^−/−^ ventricular septal defect (5/6 E18.5); *Ing4* ^+/−^;*Ing5* ^−/−^ severe malformations (from E10.5); *Ing4* ^−/−^;*Ing5* ^−/−^ E8.5 arrest	*Ing4* ^−/−^;*Ing5* ^−/−^ MEFs lose H3K14ac (HBO1 mark); E10.5 *Ing4* ^+/−^;*Ing5* ^−/−^	Redundant for E8.5 survival; ING5 dosage-critical for septation (ING4 contributes, cannot substitute)	Wu et al. (2020), Mah et al. (2024), Kueh et al. (2011), Voss et al. (2012), Vanyai et al. (2015)
Fetal liver/blood - mouse (ING5)	*Ing5* germline KO	E14.5 fetal liver 53% cellularity; ↓HSC, ↓HPC-2; erythroid maturation delay (↑proerythroblasts, ↓mature RBC); splenic hypoplasia; milder than MOZ/HBO1 loss. Competitive transplant normal → defect cell-extrinsic	MOZ/MORF + HBO1 (ING5 subunit); defect from fetal microenvironment (spleen)/developmental delay, not stem-cell-intrinsic	ING4 compensation proposed (untested)	Mah et al. (2023)
Adult bone marrow - mouse (ING4)	*Ing4* germline KO	Knockout of ING5 increased number and quiescent LT/ST-HSCs with an activated (poised) transcriptome; c-Myc inhibition releases quiescence → ING4 a negative regulator of HSC quiescence/self-renewal. MPPs fewer, poised, out-compete wild-type on competitive transplant (↑chimerism)	HBO1/BRD1 (ING4 reader); c-Myc (via AUF1)	ING4 vs. ING5 specialized (different stages); both in HBO1 → possible mutual substitution (untested)	Thompson et al. (2024), Anderson et al. (2025)
*Drosophila*	*Ing5* deletion alleles. RNAi; ectopic Ing5 overexpression (no growth effect)	*Ing5* required for differentiation/fate, not proliferation; null homozygotes arrest at prepupal stage and die; clones show antenna-to-leg transformation, disorganized ommatidia, thorax cleft, extra wing veins; Ing5/Enok complex regulates development via playing a role in controlling E Yki and Egrf pathways	Tctp binds Ing5 PHD and holds it cytoplasmic; Tctp loss → nuclear Ing5 → Enok (MOZ/MORF homolog) → H3K23ac; Ing5 represses EGFR, supports Yorkie	No fly ING4; fly Ing5 acts through Enok (MOZ/MORF) only, not the HBO1 homolog	Kim et al. (2023)

### 5.1 ING5 in differentiation of embryonic stem cells

In mouse embryonic stem cells (mESCs), ING5 restrains differentiation rather than being required to maintain the pluripotent state. ING5 mRNA and protein declined as E14, R1, and D3 mESC cell lines upon differentiation (Wang et al., 2018). In human cells ING5 protein levels decreased as embryonic stem cells differentiated into trophoblast as determined by immunofluorescence, and were undetectable in first-trimester placental trophoblast. However, mass spectrometry identified ING5 as upregulated, a discrepancy the authors did not address, treating the immunostaining as more definitive because it matched primary placental tissue (Sarkar et al., 2016). An embryoid body induction study showed that *Ing5*-KO cells formed embryoid bodies that were approximately 27% larger than those formed by wild-type cells. The loss of ING5 in mESCs reduced the levels of the pluripotency markers *Sox2*, *Nanog*, *Oct4*, and *Klf4*. However, the drop in their levels was insufficient to induce differentiation on its own (Cenik et al., 2022). Taken together, these results indicate that although ING5 may play a role in restraining ESC differentiation, the extent of its effect may vary by context.

Evidence for a potential mechanistic role for ING5 in embryonic stem cells comes from Cenik et al. (2022) who identified *Ing5* as the top hit in a genome-wide CRISPR/Cas9 dropout screen in mESCs with the H3K4 methyltransferase Set1A lacking SET (*Set1A* ^*ΔSET*^), a catalytic domain required for embryonic development. *Ing5* knockout left cells viable with minimal transcriptomic change, and an *Ing5-KO*;*Set1A* ^*ΔSET*^ double mutant survived while growing slightly slower. Because ING5 was the top dropout hit, its loss was expected to cripple *Set1A* ^*ΔSET*^ cells, so the survival of the double mutant phenotype was unexpected. The authors called the interaction a synthetic perturbation rather than a synthetic lethality on that basis. They attributed the double mutant’s survival to redundancy with ING4, though ING4 was inferred rather than tested. *Ing4* was never deleted, and the residual ING5 signal in the knockouts was assigned to antibody cross-reaction with ING4 rather than measured directly.

Cenik et al. (2022) also showed that ING5 and SET1A co-occupy about a fifth of their peaks based on ChIP-seq, and that deleting ING5 alone, or together with the SET domain on SET1A, did not significantly change global SET1A occupancy. Thus, the authors concluded that ING5 is not required for SET1A recruitment to chromatin. Additionally, among these SET1A/ING5 co-occupied promoters, the genes upregulated in the double mutant relative to wild-type showed the highest SET1A occupancy and increased development and morphogenesis, whereas the downregulated genes showed the lowest. These observations suggest that ING5 directs SET1A to specific targets on chromatin rather than recruiting SET1A to them (Cenik et al., 2022), though the mechanism remains unresolved.

Acetylation changes upon the loss of ING5 in mESCs were limited. Mass spectrometry revealed 30% and 20% reductions in BRPF1/2/3-associated HBO1 and MOZ/MORF targets H3K14ac and H3K23ac in the double mutant compared to the wild type. However, ChIP-seq confirmed only the loss of H3K23ac, leaving room for an enzyme-independent function of ING5. The JADE1/2/3-associated HBO1 targets H4K5ac, H4K8ac, and H4K12ac remained largely unchanged (Cenik et al., 2022). One possible reason for these observations is that ING4 can substitute for ING5 in HBO1/JADE/1/2/3 complex but not in MOZ/MORF/BRPF/1/2/3, so HBO1/JADE/1/2/3 acetylation may stay relatively intact when ING5 is lost.

### 5.2 ING4 roles in adult non-cancerous stem cell models

In several adult somatic stem cell types, ING5 has primarily been described as a factor that helps maintain the undifferentiated state (Mulder et al., 2012; Al Shueili et al., 2025), while the role of ING4 has been largely uncharacterized. In primary human epidermal stem cells (keratinocytes), an unbiased siRNA screen of 332 chromatin factors identified ING5 as part of a genetic interaction network with SMARCA5, BPTF, EZH2, and UHRF1, which act together to maintain keratinocytes in an undifferentiated, self-renewing state. The ChIP-seq and ChIP-qPCR analyses showed that ING5 localized to H3K4me3-marked active promoters including the basal-anchoring integrins ITGA6 and ITGB1, whose levels decrease as epidermal cells differentiate. The ChIP-qPCR experiment showed stronger enrichment of ING5, SMARCA5, and BPTF signals at the ITGB1 promoter versus the ITGA6 promoter. ShRNA depletion of ING5 or other network members in reconstituted human epidermis reduced clone formation and promoted differentiation of the basal layer cells (Mulder et al., 2012). A subsequent study showed that the splicing factor ZMAT2 interacts functionally with ING5 and other members of this network, helping maintain epidermal cells in an undifferentiated state by regulating RNA splicing. Double-knockdown experiments confirmed the interaction: silencing ZMAT2 together with ING5 produced predominantly alleviating genetic interactions, as expected for factors in a shared pathway or process (Roguev et al., 2013; Tanis et al., 2018). Exons differentially spliced after ZMAT2 depletion were enriched at ING5 binding sites, tying ZMAT2-regulated splicing to ING5-marked genes (Mulder et al., 2012; Tanis et al., 2018).

A recent study in an *Ing5* ^*−/−*^ mouse demonstrated that the subventricular zone (SVZ) of the brains of *Ing5-KO* mice had reduced levels of the *Sox2* and nestin stem cell markers and increased levels of doublecortin-positive newly differentiated neurons compared to the SVZ of wild-type mice (Al Shueili et al., 2025). Interestingly, MORF was previously identified as a marker of neural stem cells (NSCs), highly expressed in subventricular zone cells with multipotent characteristics (Sheikh et al., 2012). Likewise, MOZ was found to play an essential role in self-renewal of NSCs via *p16* ^*INK4α*^ repression (Perez-Campo et al., 2014). Because ING5 is a stoichiometric subunit of both complexes, its role in neural stem cell maintenance may reflect its function within MOZ and MORF, rather than an independent activity. However, this hypothesis may be difficult to test directly.

In mesenchymal stem cells (MSCs), siRNA knockdown of ING5 increased proliferation and raised CDK2 and PCNA expression. ING5 was found to be a direct target of miR-193, and miR-193 overexpression reduced ING5 mRNA and protein in MSCs (Wang et al., 2012). However, since miR-193 has other predicted proliferation targets, whether ING5 is a primary target through which it regulates MSC proliferation warrants further investigation.

The roles of ING4 and ING5 in Hematopoietic Stem Cells (HSCs) and their relevance for blood development are outlined in Section 5.3.2 of this review.

### 5.3 ING4/5 in mouse development

HBO1, MOZ, and MORF KATs play critical roles in mouse embryonic development (Thomas et al., 2000; Voss et al., 2009; Kraft et al., 2011; Kueh et al., 2011; Voss et al., 2012; Vanyai et al., 2015; Vanyai et al., 2019; Bergamasco et al., 2025). Thus, since ING4 and ING5 are subunits of these acetyltransferases that have already been implicated in regulating differentiation across various cell lines, their potential roles in embryonic development have also been investigated, mostly through knockout studies.

*Ing4* ^*−/−*^ mice were viable and developed normally. The phenotype was characterized by heightened sensitivity to lipopolysaccharide and increased activation of the NF-κB pathway (Coles et al., 2010). On the other hand, the phenotype of the Ing5-null mice appears more abnormal, even though details vary across reports. One study showed that *Ing5* ^*−/−*^ mice were viable but subfertile and had smaller litter sizes than wild-type animals. The proportion of *Ing5* ^*−/−*^ pups was below Mendelian expectations (∼12% versus ∼25%), which the authors attributed to prenatal death (Al Shueili et al., 2025). In a different study, an independently developed *Ing5* ^*−/−*^ line demonstrated reduced survival, with two-thirds of the pups dying between birth and weaning. The surviving mice were, however, normal and fertile. The differences between the two lines may reflect their distinct null alleles: a CRISPR frameshift in exon 3 (Al Shueili et al., 2025) versus a Cre-mediated deletion of exons 3-5, which encodes the NLS domain (Mah et al., 2023). Interestingly, *Ing5* ^*−/−*^ mice from both groups displayed blood abnormalities. While Mah et al. (2023) reported impaired hematopoiesis, Al Shueili et al. (2025) showed that mice lacking ING5 developed Diffuse Large B-cell Lymphoma (DLBCL) more frequently with aging than wild-type animals. The role of ING5 in blood development is explored in Section 5.3.2.

When *Ing4* and *Ing5* were deleted together, the phenotype was lethal due to developmental arrest at E8.5. *Ing4* ^*+/−*^ *;Ing5* ^*−/−*^ embryos survived longer but exhibited cardiac abnormalities, a collapsed rhombencephalon, smaller body size and eyes, and excess edema (Mah et al., 2024). Since the single *Ing4* and *Ing5* knockouts survived embryonic development (Coles et al., 2010; Mah et al., 2023; Mah et al., 2024; Al Shueili et al., 2025), ING4 and ING5 likely compensate for each other during embryonic development.

Voss et al. (2009) showed that knockout of MOZ in mouse embryos resulted in a decrease in ING5 occupancy in the *Hoxa* and *Hoxb* loci, locus-specific H3K9 hypoacetylation (global H3K9ac and local H3K14ac unchanged), reduced *Hox* expression, and body segment development defects. Retinoic acid restored H3K9 acetylation and the segment-identity defect but not ING5 recruitment. Because the rescue proceeded without MOZ and without ING5 at the *Hox* loci, it leaves open the question of whether the normal, MOZ-dependent H3K9 acetylation and Hox transcription activation also requires ING5, which the study did not test. To investigate it, H3K9 acetylation at *Hox* loci, *Hox* gene expression, and retinoic acid rescue could be assessed directly in *Ing5* ^*−/−*^ embryos with MOZ intact.

#### 5.3.1 ING4/5 in cardiac development

Previously, it was reported that in the postnatal heart, ING5 restrains cardiomyocyte proliferation in a p53-independent manner by maintaining p21 levels, and that its mRNA levels are stabilized by LRP6, a critical regulator of heart development (Wu et al., 2020). The roles of ING4 in cardiomyocyte proliferation have not been explored.

However, earlier in heart development, ING4 and ING5 were found to compensate for each other. A germline *Ing4*; *Ing5* allelic series revealed that in mouse embryos this compensation was graded rather than all-or-none. Although Mah et al. (2024) did not describe the hearts of the single *Ing4* ^*−/−*^ *; Ing5* ^*+/+*^mice, the overall phenotypes appeared largely normal (Coles et al., 2010; Mah et al., 2024). *Ing4* ^*+/+*^ *; Ing5* ^*−/−*^ mice had isolated ventricular septal defects in 5/6 E18.5 fetuses. Removing one further *Ing4* allele led to heart anomalies observed from E10.5 and fully penetrant by E13.5. *Ing4* ^*+/−*^ *; Ing5* ^*−/−*^ embryos showed abnormalities including septal defects, overriding aorta, atrioventricular cushion remodelling defects, indistinct interventricular sulcus, misshapen apex, thin compact myocardium, malformed valves, and excessive epicardial blebbing (Mah et al., 2024). Loss of all four alleles in *Ing4* ^*+/+*^ *; Ing5* ^*−/−*^ embryos led to failure of chorioallantoic fusion and developmental arrest at E8.5, phenocopying the loss of HBO1 (Kueh et al., 2011; Mah et al., 2024). Additionally, *Ing4* ^*−/−*^ *;Ing5* ^*−/−*^ MEFs lost H3K14ac (Mah et al., 2024), the mark lost in *Hbo1* ^*−/−*^ embryos (Kueh et al., 2011). Hence, this role is likely to be attributable to HBO1.

The allelic series noted above separates the roles of the ING paralogs. ING4 and ING5 are redundant for early embryonic survival: a single allele of either gene is sufficient to prevent the E8.5 arrest. Septation is the one context where the paralogs are not equivalent. ING5 is dosage-critical: full *Ing4* ^*+/+*^ did not prevent the *Ing5* ^*−/−*^ septal defect, and removing one *Ing4* allele worsened the cardiac phenotype, so ING4 contributes to cardiac development without substituting the role of ING5. The *Ing5* ^*−/−*^ defects were similar to those observed in the heart phenotypes of the *Moz* ^*+/−*^ mice (Voss et al., 2012; Vanyai et al., 2015) and humans (Kennedy et al., 2019). Additionally, in E10.5 hearts, the *Ing4* ^*+/−*^ *;Ing5* ^*−/−*^ transcriptome positively correlated with that of *Moz* ^−/−^ (P = 0.001), consistent with ING5 acting within the MOZ complex (Mah et al., 2024). The cardiac defects were more severe in *Ing4* ^*−/−*^ *;Ing5* ^*−/−*^ than in *Moz* ^−/−^ (Voss et al., 2012), indicating that MORF and HBO1 complexes may also contribute to the anomalies in the hearts of the *Ing4* ^*−/−*^ *;Ing5* ^*−/−*^ embryos.

#### 5.3.2 ING4/5 in blood development

MOZ/MORF and HBO1 are central to blood formation, making ING4 and ING5 candidate regulators of hematopoiesis. MOZ is required for the development and maintenance of long-term reconstituting HSCs, as *Moz*-mutant and *Moz*-null fetal liver cells fail to reconstitute irradiated recipients, a defect intrinsic to the stem cell compartment (Thomas et al., 2006; Katsumoto et al., 2006). Mouse embryos lacking the catalytic domain of MOZ also had a lower number of HSCs. They exhibited hindered B-cell development, demonstrating the importance of its acetyltransferase activity in blood development (Perez-Campo et al., 2009).

HBO1 was found to support HSC quiescence. Conditional *Hbo1* deletion drove quiescent HSCs into the cell cycle, resulting in dividing stem cells that were producing progenitors rather than self-renewing. The HSC pool was depleted, with the loss of H3K14 acetylation across the self-renewal network, including *Tek*, *Mpl*, *Gata1*, *Gata2*, *Egr1*, *Stat5a*, *Pbx1*, *Meis1*, *Gfb1*, and *Hoxa* genes (Yang et al., 2022). HBO1 is also required for fetal liver erythropoiesis: the HBO1/BRD1 complex was found to deposit global H3K14 acetylation. Mice embryos deficient in *Brd1* had reduced levels of *Gata1* and other erythroid regulators, arrested erythroblast maturation, and died at mid-gestation from lethal fetal anemia (Mishima et al., 2011).

A germline deletion of *Ing5* produced a phenotype milder than that observed in embryos deficient for *Moz* or *Hbo1* (Thomas et al., 2006; Katsumoto et al., 2006; Yang et al., 2022; Mah et al., 2023). The fetal liver cellularity at E14.5 was 53% of wild-type levels, indicating reduced liver size. Within the fetal liver, HSCs and the hematopoietic progenitor cell 2 (HPC-2) populations were reduced. Erythropoiesis stalled, with an increase in proerythroblasts and a loss of mature erythrocytes and the spleen was underdeveloped. The erythroid changes resembled the maturation delay caused by loss of MOZ or HBO1, with milder hypoplasia than in *Moz*-null fetuses (Thomas et al., 2006; Yang et al., 2022; Mah et al., 2023), potentially suggesting compensatory effects of ING4. A stronger contrast emerged in transplantation. However, unlike the loss of HBO1 or MOZ, the deletion of ING5 did not alter the capacity to repopulate irradiated recipient mice. Mah et al. (2023) concluded that the defect is likely to reflect a developmental delay or altered fetal microenvironment, particularly in the spleen. Contrary to the hypoplasia observed in the *Ing5* ^*−/−*^associated phenotype, another study reported that the loss of ING5 resulted in enlarged spleen and lymph node in mice. However, this was only observed in mice who were 18 months old or older. It was likely due to increased instances of DLBCLs (Al Shueili et al., 2025) rather than a direct result of impaired hematopoiesis.

Although the role of ING4 in hematopoiesis during embryonic development has not yet been established, it has been found to play a distinct role in adult murine HSCs. In adult *Ing4* ^*−/−*^ bone marrow, both long-term and short-term HSCs were more numerous and quiescent. Nevertheless, their transcriptional profile was characteristic of an activated, poised HSC (Thompson et al., 2024). These HSCs remained functional upon transplantation, and their quiescence was disrupted by inhibiting c-MYC, a downstream target of ING4 via its interaction with AUF1 (Lu et al., 2013a), thereby identifying ING4 as a negative regulator of HSC quiescence and self-renewal (Thompson et al., 2024). The same poised state was observed in multipotent progenitors (MPPs) upon the loss of ING4. In the bone marrow of young *Ing4* ^*−/−*^ mice, there were fewer MPPs than in the bone marrow of age-matched wild-type mice. Moreover, the remaining MPPs were additionally more poised. They had greater regenerative capacity than wild-type MPPs and led to higher blood chimerism following competitive transplantation (Anderson et al., 2025).

Taken together, ING4 and ING5 may act at different stages of blood development. ING4 acts to restrain HSC and progenitor self-renewal and activation, so that its loss leaves these cells poised for stronger regeneration. ING5, by contrast, supports fetal HSC numbers and erythropoiesis. However, given that both can be subunits of HBO1, a KAT shown to play a critical role in blood development (Yang et al., 2022), they may also substitute for each other. Allele series of *Ing4*/*Ing5* knockouts in blood cells would help evaluate this possibility.

### 5.4 Ing5 roles in *Drosophila* development

The developmental roles of ING5 have extended beyond mammalian models. Kim et al. (2023) showed that, in *Drosophila*, Ing5 is required for proper differentiation and cell-fate determination, but not for proliferation. *Ing5*-null homozygotes were arrested at the prepupal stage and died, and adult flies bearing Ing5-null clones showed antenna-to-leg homeotic transformation, disorganized ommatidia, a thorax cleft, and extra wing veins.

The central finding was that ING5 roles were inhibited by translationally controlled tumor protein (Tctp), which bound the PHD domain of Ing5 and held it in the cytoplasm, blocking nuclear entry. When Tctp was depleted, Ing5 accumulated in the nucleus, associated with Enok (the MOZ/MORF homolog), and brought the complex onto chromatin to acetylate H3K23. Ing5 also influenced two growth pathways. Functional Ing5 repressed the Egfr pathway and promoted the Yki pathway, so the knockdown of Ing5 had the opposite effect on them, giving Ing5 mutants small wings with extra veins. The loss of Ing5 alone did not cause overgrowth, but when Yki was activated by mis-regulated Crumbs, removing both Ing5 and Enok led to tumor-like tissue overgrowth, identifying the Ing5-Enok complex as a brake on Yki-driven overgrowth. Thus, in flies, Ing5 may play a role in regulating organ development by regulating Yki, ensuring it is as active as needed for normal development (Kim et al., 2023). To our knowledge, no work describing Ing4 in *Drosophila* or other invertebrates has been published.

## 6 Conclusion

ING4 and ING5 are the closest paralogs in the ING family and act primarily as tumor suppressors, but neither is uniformly so, and both play roles in differentiation and development. The two proteins can compensate for each other within the HBO1 complex, whereas only ING5 associates with the MOZ/MORF complexes. Shared membership allows redundancy; ING5’s additional partners may give it broader non-overlapping roles.

In cancers, the paralogs converge on the p53, Wnt/β-catenin, and to some extent the NF-κB pathways and diverge via other mechanisms. When they act as tumor suppressors, their loss in cancers is generally driven by reduced expression or mislocalization rather than by mutation. Both proteins were found to turn pro-malignant in stem-like contexts: ING5 in BTICs and ING4 in RCC, promoting self-renewal rather than restraining it (Figure 5).

**FIGURE 6:**
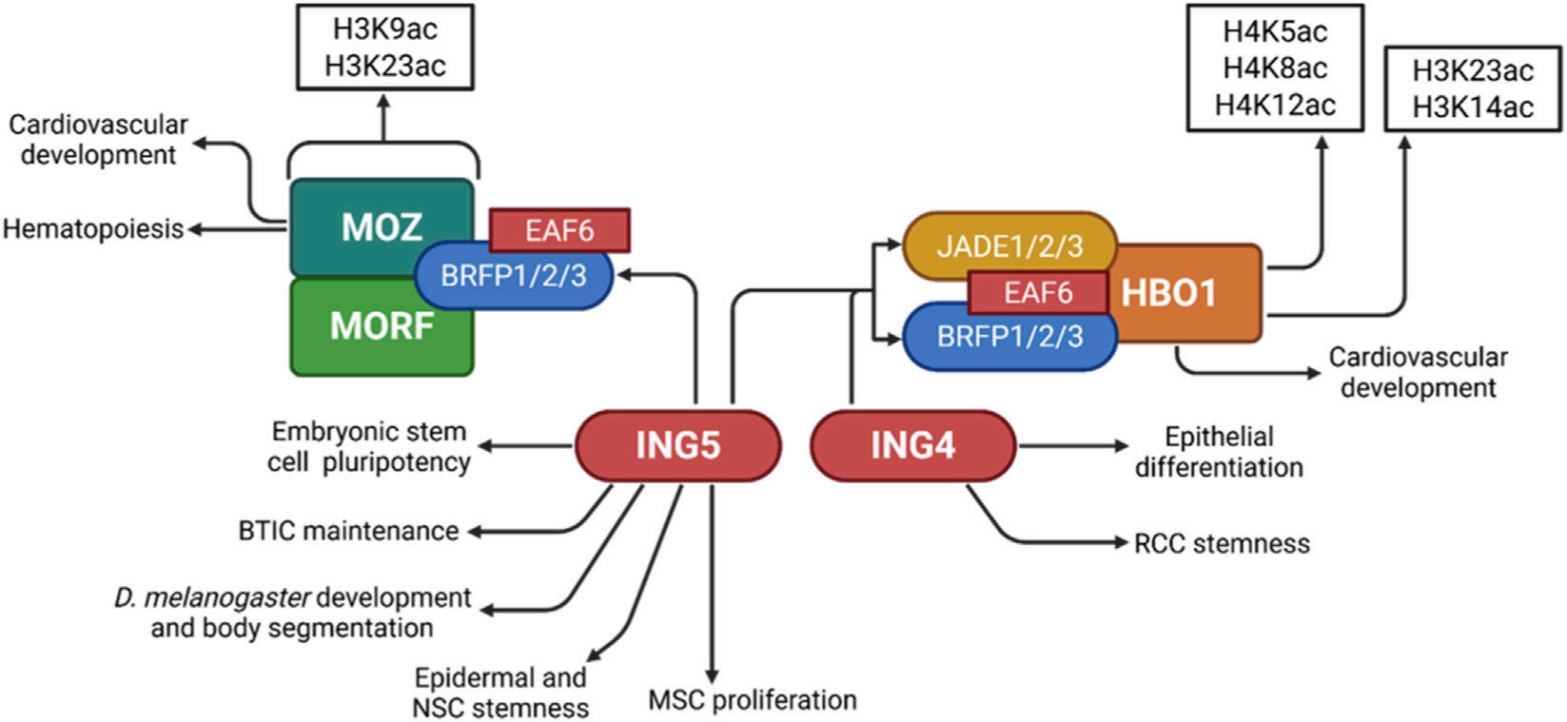
ING4 and ING5 in development and stemness. Both ING paralogs have been associated with aspects of development or stem cell maintenance. ING4 and ING5 independently associate with HBO1, connected to the complex by binding the JADE/BRPF scaffold. Through HBO1, both ING4 and ING5 have been linked to cardiovascular development (Mah et al., 2024). ING4 has independently been associated with promoting stem-like properties and proliferation in renal cell carcinoma cells (RCC’s) and epithelial cell cultures (Tang et al., 2022). Between the two paralogs, ING5 has been more broadly associated with stemness; promoting growth, pluripotency or maintenance of stemness markers in various cell lines, including mesenchymal stem cells (MSCs), embryonic stem cells, epidermal and neural stem cells, and glioblastoma associated BTICs (Wang et al., 2012; Wang et al., 2018; Cenik et al., 2022; Al Shueili et al., 2025). Beyond these, ING5 has been linked to hematopoiesis and cardiovascular development through its association with MOZ/MORF, while a role in body segmentation and patterning in *Drosophila* has also been reported (Kim et al., 2023; Mah et al., 2023; Mah et al., 2024). HBO1 and MOZ/MORF complexes have each been linked to specific acetylation patterns, likely contributing to their modulation of genes associated with development, cell survival, proliferation and maintenance. Although histone specificity appears to vary based on the associated scaffold, ING4/5 mutations have been linked to reductions in the levels of specific HBO1-MOZ/MORF associated acetylation marks (Mah et al., 2024).

In differentiation and development, ING5 has been characterized much more extensively than ING4. However, both were found to maintain stemness and normal development in mice, sometimes compensating for each other’s loss. Double knockout of both paralogs leads to severe defects in the heart and developmental arrest early in embryonic development. However, the presence of either of them significantly increases mouse survival (Figure 6).

In future studies, graded Ing4/Ing5 allele series in defined tissues, particularly blood and heart, would map where the paralogs compensate and where they do not. Endogenous loss-of-function and *in vivo* work would test the ING4 and ING5 cancer mechanisms that overexpression alone cannot establish. The structural basis for ING5’s selectivity toward MOZ/MORF remains an open and interesting question. Determining whether both paralogs promote stemness across cancers, and whether they are interchangeable in this context, would also indicate whether they are worth pursuing as targets in cancer stem cells. Finally, since it has been established that ING5 participates in regulation and development in both mammals (humans and mice) and invertebrates (flies), research on ING4/5 could benefit from investigating their roles in other species, particularly in development.
